# Surgical complications after immediate implant-based breast reconstruction for breast cancer in women over 65 years

**DOI:** 10.1093/bjsopen/zrae095

**Published:** 2024-10-09

**Authors:** Yihang Liu, Anna L V Johansson, Ira Oikonomou, Axel Frisell, Hannah C Adam, Dhirar Ansarei, Martin Halle, Helena Sackey, Jana de Boniface

**Affiliations:** Department of Molecular Medicine and Surgery, Karolinska Institutet, Stockholm, Sweden; Department of Acute and Trauma Surgery, Karolinska University Hospital, Stockholm, Sweden; Department of Medical Epidemiology and Biostatistics, Karolinska Institutet, Stockholm, Sweden; Cancer Registry of Norway, Oslo, Norway; Department of Surgery, South General Hospital, Stockholm, Sweden; Department of Dermatology and Venerology, Karolinska University Hospital, Stockholm, Sweden; Department of Orthopaedic Surgery, Danderyd Hospital, Stockholm, Sweden; Department of Surgery, Västmanlands sjukhus Västerås, Västerås, Stockholm, Sweden; Department of Molecular Medicine and Surgery, Karolinska Institutet, Stockholm, Sweden; Department of Reconstructive Plastic Surgery, Karolinska University Hospital, Stockholm, Sweden; Department of Molecular Medicine and Surgery, Karolinska Institutet, Stockholm, Sweden; Department of Breast Cancer, Endocrine Tumours and Sarcoma, Karolinska University Hospital, Stockholm, Sweden; Department of Molecular Medicine and Surgery, Karolinska Institutet, Stockholm, Sweden; Department of Surgery, Capio St Göran’s Hospital, Stockholm, Sweden

## Abstract

**Background:**

While immediate breast reconstruction rates in breast cancer are increasing, they remain low in women over 65 years old. The aim was to investigate surgical outcomes in women older than 65 years receiving implant-based immediate breast reconstruction.

**Method:**

The population-based Stockholm Breast Reconstruction Database includes all adult women with breast cancer receiving an implant-based immediate breast reconstruction in Stockholm, Sweden, 2005–2015. Primary outcomes within 30 days from immediate breast reconstruction were: infection requiring antibiotics and reoperation on. Implant removal was a secondary outcome. Women more than 65 years were compared with younger age groups. Chi-square tests and multivariable logistic regression were applied for the primary outcomes, and Kaplan–Meier analysis for the secondary outcome.

**Results:**

Among 1749 cases of immediate breast reconstruction, 140 (8.0%) were in women more than 65 years. Median follow-up was 74 months (1–198). Postoperative infection was not more common in women older than 65 years old (22 of 140, 15.7%) than in women under 65 years old (303 of 1609, 18.8%; *P* = 0.221). Reoperation on was more frequent in women older than 65 years than in other age groups (more than 65: 8.6%; 50–64: 6.5%; 40–49: 3.5%; less than 40: 1.6%; *P* < 0.001), however, age older than 65 years was not an independent risk factor in the multivariable analysis (OR 1.00, 95% c.i. 0.44 to 2.28). Overall, 6-year probability of implant removal was 11.4%, (8.1% due to complications and 3.3% due to patient preference). There was no statistically significant difference between age groups for either reason (*P* = 0.085 and *P* = 0.794 respectively).

**Conclusion:**

Older age alone was not associated with worse surgical outcomes after implant-based immediate breast reconstruction in highly selected patients older than 65 years when compared with their younger counterparts.

## Teaser

The study investigates surgical outcomes in women aged 65 years and older undergoing implant-based immediate breast reconstruction. Despite a higher rate of reoperation on in this age group, age was not found to be an independent risk factor for postoperative complications. The findings suggest that implant-based immediate breast reconstruction is safe for selected women aged 65 and older.

## Introduction

Despite the notion that breast conservation results in better survival, mastectomy rates are still considerable^1^. In patients having undergone a mastectomy, breast reconstruction should be discussed. The choice of reconstructive strategy is guided by patient and disease factors, surgeon expertise and training, local routines, expected postoperative treatment and patient preference. In Sweden, immediate breast reconstruction (IBR) is most commonly implant based, probably due to logistic challenges and limited microsurgical resources when considering autologous IBR options. In recent years, overall rates of IBR in Sweden have increased from 6% in 2008 to 16% in 2022^2^.

Risk factors for complication in reconstructive breast surgery include body mass index (BMI), extent of disease and surgery, use of foreign material and postmastectomy radiation^3,4^. Even though several studies have shown that age is not an independent risk factor for surgical complications in reconstructive breast surgery^5,6^, IBR is rarely offered to older women^7,8^. This is likely due to multiple factors; for example the higher proportion of co-morbidities and thus potential contraindications to reconstructive surgery in older women^9^, the smaller proportion of older women rating breast reconstruction as important, but also surgeons’ conceptions of risk levels in older individuals and the potential assumption that aesthetics are less important for older women^9,10^. The life expectancy of patients with breast cancer has increased during the last decades, raising issues of survivorship and health-related quality of life (HRQoL)^11^. For women who have undergone a mastectomy, studies report improved HRQoL and body image when breast reconstruction is performed^12–14^. This was also true when older ages were specifically included in the study population^15^.

In Sweden, 25% of patients under 65 years who had a mastectomy received implant-based IBR in 2021 and 31% in 2022, but only 2% of patients more than 65 years in both 2021 and 2022^2^. Furthermore, in the USA and the UK, breast reconstruction is rarely performed in patients in higher age groups^16–18^.

This study aimed to investigate 30-day postoperative complication rates and long-term surgical outcomes in women aged more than 65 years who received implant-based IBR.

## Methods

### Study design

This study utilizes the population-based Stockholm Breast Reconstruction Database version 1.0, which has been previously described in detail^19^. In summary, all women with invasive or *in situ* breast cancer undergoing therapeutic mastectomy and implant-based IBR at one of the four breast cancer centres in Stockholm, Sweden, from 1 January 2005 to 31 December 2015 were identified from the Swedish National Patient Register by their ICD (C509, D051) and procedure codes (HAC combined with HAE). The National Patient Register includes all in-patient care episodes in Sweden and has national coverage since 1987. Information includes diagnosis and procedure codes (according to ICD 10), date of admission and discharge as well as individual personal identification numbers, which enable individual-level linkage and retrieval of medical charts. All identified cases were first checked against medical charts to only select implant-based IBR and then registered in an electronic Case Report Form (eCRF).

Bilateral cases were included if both sides fulfilled the inclusion criteria. Both primary and recurrent breast cancers were eligible. Medical charts were individually reviewed by a team of dedicated breast surgeons and trainees during dates with the last update on 21 November 2021. Tumour and patient characteristics, oncological treatment, detailed data on surgical procedure, reconstructive and revisional interventions, risk factors and complications, as well as oncological follow-up and death were recorded. Patient weight was divided into four groups using BMI, based on the classification from the World Health Organization: <18.5 (underweight), 18.5–24.9 (normal weight), 25.0–29.9 (preobesity) and >30 (obesity)^20^. Further risk factors included use of antihypertensive, immunosuppressive or antidiabetic medication (peroral treatment *versus* insulin-dependent diabetes) and previous or current smoking. For completeness, data were also cross-linked with the Swedish National Quality Register for Breast Cancer (NKBC) and historical breast cancer registries^21^.

The two primary outcomes were extracted from medical charts by individual scrutiny: postoperative infection within 30 days after IBR was defined as a suspected or confirmed infection requiring oral or intravenous antibiotic treatment, and reoperation on was defined as any unplanned return to theatre for any surgical complication within 30 days of IBR. Individuals with mild clinical signs of infection who did not receive antibiotic treatment were categorized as having no infection. In the Stockholm region, all patients are followed up at their breast unit after surgery. Even if the patient first visits emergency care or primary healthcare, any review and follow-up is always conducted at the breast unit; therefore, capture of infection rates is deemed excellent. The secondary outcome, implant removal, was extracted by scrutiny of the entire medical chart and was defined as removal of the breast implant at any time during follow-up with or without simultaneous replacement with an autologous reconstructive option. Implant exchange did thus not count as implant removal. Follow-up for implant removal was defined as time from IBR to implant removal or to last date of contact with a physician or nurse according to the medical chart. Data on conversion to autologous reconstruction were also collected from medical charts. Breast implants were categorized into three groups; permanent implants (fixed-volume silicone implants), permanent expanders (combination of silicone and saline filling, no mandatory exchange to implant) and temporary expanders (saline filling only, mandatory exchange to implant). Revision surgery was defined as any ipsilateral surgical procedure not removing the implant (implant exchange, capsulectomy) but entering the implant cavity; thus, lipofilling and nipple reconstruction were not counted as revision surgery.

Ethical permission was obtained from the Swedish Ethical Review Authority (2015/1183-31/4), including three amendments (2016/1374-32; 2017/2318-32; 2018/42-32). In accordance with the ethical permission and the additional regulatory review by each hospital director, individual consent for clinical data extraction from medical charts and registration was not deemed necessary at the time.

### Statistical analysis

Age at IBR was categorized into four groups: under 40, 40–49, 50–64 and older than 65 years. Descriptive statistics of clinical variables by age are presented as numbers with percentages, mean values with standard deviations, or median values with minimum and maximum values. All presented variables are per breast/IBR. For comparison of categorical variables by age group, the chi-square or Fisher’s exact test were used as appropriate, while the Kruskal–Wallis test was used for comparison of continuous variables by age group. The associations of potential clinical risk factors with the primary outcomes were assessed by logistic regression models and the results are presented as odds ratios (OR) with 95% confidence intervals (c.i.). First, univariable models were estimated. The subsequent multivariable models included the covariates: previous radiotherapy (RT), BMI, smoking status, hypertension and the surgical variables: specimen weight as a proxy for breast size, type of breast implant and nipple-sparing as opposed to skin-sparing mastectomy. Diabetes mellitus and immunosuppressive medication were only entered into univariable analyses due to the limited number of cases and events. Both uni- and multivariable analyses were based on complete cases only, that is cases with missing values in any of the selected covariates were not included in either of the analyses.

For the secondary outcome, probabilities of implant removal were estimated as one minus the survival proportion from Kaplan–Meier analysis. Due to high survival rates and relatively short follow-up, we did not incorporate competing risks of death.

IBM Statistical Package for the Social Sciences (SPSS) for Macintosh (Version 28.0. Armonk, NY: IBM Corp) was used for all analyses.

## Results

In total, 1687 women receiving 1749 implant-based IBRs were included. The age distribution of the 1749 occurrences of IBR was as follows: 255 IBRs (14.6%) under 40 years of age, 687 (39.3%) 40–54 years, 667 (38.1%) 55–64 years and 140 (8.0%) older than 65 years of age. Median follow-up time was 74 months (1–198). At the end of follow-up, 167 women were deceased (representing 177 IBRs). Women more than 65 years selected for IBR more often had non-invasive disease, lower tumour stages, a higher proportion of lobular histology, less extensive axillary surgery and more favourable tumour biology (*Table 1*). Accordingly, lower rates of adjuvant RT, primary systemic therapy, adjuvant chemotherapy and targeted anti-human epidermal growth factor receptor (HER) 2 treatment were observed. Hypertension was generally rare but most common in women more than 65 years, while diabetes mellitus was more common in women above 50 years old (*Table 2*). While the use of acellular dermal matrix (ADM) or synthetic mesh was uncommon and did not differ between age groups (*P* = 0.890), it increased over time from 1 patient in 2008 (1.0%) to 34 patients in 2015 (17.0%).

**Table 1:** Patient, treatment, and disease characteristics in 1749 occurrences of IBR in 1687 women 2005–2015 in Sweden

	<40 years *N* = 255	40–49 years *N* = 687	50–64 years *N* = 667	≥65 years *N* = 140	*P*
**Calendar interval for IBR (%)**					
2005–2007	45 (17.6)	115 (16.7)	185 (27.7)	24 (17.1)	<0.001
2008–2010	80 (31.4)	180 (26.2)	190 (28.5)	45 (32.1)	
2011–2013	60 (23.5)	227 (33.0)	173 (25.9)	39 (27.8)	
2014–2015	70 (27.4)	165 (24.0)	119 (17.8)	32 (22.8)	
Follow-up (months)*	77 (2–197)	72 (1–191)	72 (1–182)	70 (1–149)	0.281
**Invasiveness (%)**					<0.001
*In situ* only	33 (12.9)	146 (21.3)	171 (25.6)	32 (22.9)	
Invasive	222 (87.1)	541 (78.7)	496 (74.4)	108 (77.1)	
**Tumour multifocality (%)†**					0.284
Unifocal	137 (61.7)	308 (56.9)	311 (62.7)	67 (62.0)	
Multifocal	81 (36.5)	225 (41.6)	180 (36.3)	41 (38.0)	
*Missing*	4 (1.8)	8 (1.5)	5 (1.0)	0 (0.0)	
**Histological tumour type (%)†,‡**					<0.001
Ductal	194 (87.4)	430 (79.5)	372 (75.0)	72 (66.7)	
Lobular	13 (5.9)	68 (12.6)	91 (18.3)	24 (22.2)	
Mixed	6 (2.7)	22 (3.8)	19 (3.8)	8 (7.4)	
Other	9 (4.1)	21 (3.9)	14 (2.8)	4 (3.7)	
**Invasive tumour stage (%)†,‡**					<0.001
T1	99 (44.6)	286 (52.8)	287 (57.8)	64 (59.2)	
T2	98 (44.1)	194 (35.8)	175 (35.3)	34 (31.5)	
T3	22 (9.9)	56 (10.3)	33 (6.6)	10 (9.2)	
Missing	3 (1.3)	5 (0.9)	1 (0.2)	0 (0)	
Invasive tumour size (mm)*,§	23 (0.5–90)	17(1–130)	17 (1–100)	17 (1–70)	0.655
**Clinical nodal stage (%)**					<0.001
cN0	207 (81.2)	609 (88.6)	605 (90.7)	129 (92.1)	
cN1	47 (18.4)	67 (9.8)	46 (6.9)	10 (7.1)	
*Missing*	1 (0.4)	11 (1.6)	16 (2.4)	1 (0.7)	
**Pathological nodal stage (%)§,¶**					0.426
pN0	123(68.3)	406 (72.2)	408 (74.3)	87 (75.7)	
pN1	45 (25.0)	122 (21.7)	104 (18.9)	24 (20.9)	
pN2	7 (3.9)	27 (4.8)	27 (4.9)	2 (1.7)	
pN3	5 (2.8)	6 (1.1)	7 (1.3)	1 (0.9)	
Missing	0 (0.0)	1 (0.2)	3 (0.5)	1 (0.9)	
**Axillary staging surgery (%)**					<0.001
Sentinel node biopsy only	120 (47.1)	381 (55.5)	378 (56.7)	79 (56.4)	
Axillary lymph node dissection	129 (50.6)	267(38.9)	216 (32.4)	41 (29.3)	
No surgical axillary staging	6 (2.4)	39 (5.7)	73 (10.9)	20 (14.3)	
**Nottingham Histological Grade (%)†,‡**					<0.001
Grade 1	12 (5.4)	65 (12.0)	77 (15.5)	13 (12.0)	
Grade 2	80 (36.0)	270 (49.9)	244 (49.2)	64 (59.3)	
Grade 3	119 (53.6)	187 (34.6)	164 (33.1)	27 (25.0)	
*Missing*	11 (5.0)	19 (3.5)	11 (2.2)	4 (3.7)	
**Estrogen receptor status (%)†,‡**					<0.001
Positive	155 (69.8)	451 (83.4)	407 (82.1)	92 (85.2)	
Negative	65 (29.3)	89 (16.5)	88 (17.7)	15 (13.9)	
Missing	2 (0.9)	1 (0.2)	1 (0.2)	1 (0.9)	
**Progesterone receptor status (%)†,‡**					0.205
Positive	121 (55.3)	391 (73.8)	302 (62.5)	75 (70.1)	
Negative	96 (43.8)	138 (26.0)	180 (37.3)	31 (29.0)	
Missing	5 (0.9)	12 (0.2)	14 (0.2)	2 (0.9)	
**HER2 status (%)**†,‡					0.010
Negative	151(68.0)	420 (77.6)	373 (75.2)	90 (83.3)	
Positive	63 (28.4)	104 (19.2)	101 (20.4)	16 (14.8)	
Missing	8 (3.6)	17 (3.1)	22 (4.4)	2 (1.9)	
**Tumour subtype (%)†,‡**					<0.001
HR + HER2−	110 (49.5)	375 (69.3)	337 (67.9)	82 (75.9)	
HR + HER2+	40 (18.0)	62 (11.5)	52 (10.5)	9 (8.3)	
HR−HER2−	41 (18.5)	45 (8.3)	36 (7.3)	8 (7.4)	
HR−HER2+	23 (10.4)	42 (7.8)	49 (9.9)	7 (6.5)	
Missing	8 (3.6)	17 (3.1)	22 (4.4)	2 (1.9)	
Proliferation†,‡ (Ki67 in %)#	38.7(23.4)	27.3(21.8)	25.0(20.6)	25.3(21.9)	<0.001
**Primary treatment†**					<0.001
Primary surgery	154 (69.4)	457 (84.5)	451 (90.9)	103 (95.4)	
Neoadjuvant chemotherapy	68 (30.6)	84 (15.5)	45 (9.1)	5 (4.6)	
**Radiotherapy (%)**					
No radiotherapy	102 (40.0)	305 (44.4)	369 (55.3)	80 (57.1)	<0.001
Previous radiotherapy	4 (1.5)	46 (6.7)	77 (11.5)	17 (12.1)	
Adjuvant radiotherapy	149 (58.4)	336 (48.9)	221 (33.1)	43 (30.7)	
**Chemotherapy (%)†**					<0.001
Yes	198 (89.2)	375 (69.3)	273 (55.0)	49 (45.4)	
No	22 (9.9)	161 (29.8)	216 (43.5)	58 (53.7)	
Missing	2 (0.9)	5 (0.9)	7 (1.4)	1 (0.9)	
**Endocrine treatment (%)†**					<0.001
Yes	154 (69.4)	443 (81.9)	401 (80.8)	95 (88.0)	
No	68 (30.6)	95 (17.6)	89 (17.9)	13 (12.0)	
Missing	0 (0.0)	3 (0.6)	6 (1.2)	0 (0.0)	
**Anti-HER2 targeted therapy (%)†**					0.001
Yes	55 (27.2)	92 (18.3)	76 (16.4)	11 (10.5)	
No	147 (72.8)	408 (81.1)	383 (82.7)	94 (89.5)	
Missing	20 (0.0)	41 (0.6)	37 (0.9)	3 (0.0)	

Values are *n* (%) unless otherwise indicated. Percentages may not always sum up to 100% due to rounding. *Median (minimum–maximum). †Invasive disease only. ‡Calculated based on surgical specimen in case of primary surgery, and on pretreatment core needle biopsy in case of neoadjuvant therapy. §Primary surgery only. ¶cases without any axillary staging excluded. #Mean(standard deviation). IBR, immediate breast reconstruction; cN, clinical nodal status; pN, pathological nodal status; HER, human epidermal growth factor receptor; HR, hormone receptor.

**Table 2:** Potential risk factors for negative surgical outcomes by age group

	<40 years *N* = 255	40–49 years *N* = 687	50–64 years *N* = 667	≥65 years *N* = 140	*P*
**BMI kg/m^2^ (%)**					0.020
Underweight (<18.5)	5 (1.9)	14 (2.0)	12 (1.8)	3 (2.1)	
Normal weight (18.5–24.9)	166 (65.1)	432 (62.9)	353 (52.9)	75 (53.6)	
Pre-obesity (25.0–29.9)	59 (23.1)	161 (23.4)	196 (29.4)	41 (29.3)	
Obesity (≥30.0)	9 (3.5)	32 (4.7)	44 (6.6)	11 (7.8)	
Missing	16 (6.3)	48 (7.0)	62 (9.3)	10 (7.1)	
**Nipple-sparing mastectomy (%)**					<0.001
Yes	51 (20.0)	94 (13.7)	61 (9.1)	19 (13.6)	
No	203 (79.6)	591 (86.0)	601 (90.1)	120 (85.7)	
Missing	1 (0.4)	2 (0.3)	5 (0.7)	1 (0.7)	
Mastectomy specimen weight (g)*	320 (66–1750)	335 (73–1500)	360 (67–2232)	328 (104–1362)	0.090
**Mastectomy specimen weight categories (g)**					0.074
<300	100 (39.2)	260 (37.8)	213 (31.9)	47 (33.6)	
300–499	78 (30.6)	236 (34.4)	215 (32.2)	32 (22.9)	
500 and more	55 (21.6)	125 (18.2)	149 (22.3)	33 (23.6)	
Missing	22 (8.6)	66 (9.6)	90 (13.5)	28 (20)	
**Smoking status (%)**					0.004
Non-smoker	212 (83.1)	537 (78.2)	480 (72.0)	103 (73.6)	
Active smoker	15 (5.9)	60 (8.7)	66 (9.9)	12 (8.6)	
Previous smoker	19 (7.5)	53 (7.7)	87 (13.0)	18 (12.9)	
Missing	9 (3.5)	37 (5.4)	34 (5.1)	7 (5.0)	
**Diabetes (%)**					0.036
Yes	0 (0.0)	3 (0.4)	11 (1.6)	1 (0.7)	
No	251 (98.4)	668 (97.2)	645 (96.7)	136 (97.1)	
Missing	4 (1.6)	16 (2.3)	11 (1.6)	3 (2.1)	
**Antihypertensive medication (%)**					<0.001
Yes	0 (0.0)	16 (2.3)	83 (12.4)	25 (17.9)	
No	251 (98.4)	655 (95.3)	574 (86.1)	110 (78.6)	
Missing	4 (1.6)	16 (2.3)	10 (1.5)	5 (3.6)	
**Immunosuppressive medication (%)**					0.541
Yes	5 (2.0)	13 (1.9)	7 (1.0)	3 (2.1)	
No	245 (96.1)	657 (95.6)	646 (96.9)	133 (95.0)	
Missing	5 (2.0)	17 (2.5)	14 (2.1)	4 (2.9)	
**Type of breast implant or expander device (%)**					<0.001
Permanent implant	57 (22.4)	149 (21.7)	150 (22.5)	40 (28.6)	
Permanent expander	112 (43.9)	338 (49.2)	309 (46.3)	66 (47.1)	
Temporary expander	85 (33.3)	190 (27.7)	194 (29.1)	33 (23.6)	
Missing	1 (0.4)	10 (1.5)	14 (2.1)	1 (0.7)	
**Matrix use (%)**					0.890
Yes (ADM or synthetic mesh)	13 (5.0)	27 (4.0)	28 (4.2)	6 (4.2)	
No	242 (95.0)	660 (96.0)	639 (95.8)	136 (95.8)	

Values are *n* (%) unless otherwise indicated. *Median (minimum, maximum). BMI, body mass index; ADM, acellular dermal matrix.

Within the first 30 days after IBR, postoperative infections were registered in 325 patients (18.6%): 42 (16.5%) in women under 40 years, 121 (17.6%) in women 40–49 years, 140 (21.0%) in women 50–64 years and 22 (15.7%) in women more than 65 years. There were no significant differences between age groups (*P* = 0.221). In the multivariable regression analysis, age was not associated with a higher risk of developing postoperative infection (OR 0.63, 95% c.i. 0.34 to 1.14) (*Supplementary Table S1*). Instead, higher mastectomy specimen weight, use of ADM or mesh, active smoking, and permanent breast implants as opposed to temporary expanders were associated with infection. Previous RT, preobesity and obesity were associated with a higher risk of infection in the univariable but not in the multivariable analysis.

A reoperation on for surgical complications within the first 30 days after IBR occurred in 83 patients (4.7%): 4 (1.6%) in women under 40 years, 24 (3.5%) in women 40–49 years, 43 (6.4%) in women 50–64 years and 12 cases (8.6%) in women more than 65 years old (*P* < 0.001). Infection and bleeding were the most common indications (*N* = 63, 75.9%). In the multivariable analysis (see *Supplementary Table S2*), age groups less than 40 and 40–49 years showed an independent association with a lower risk of reoperation on compared with women 50–64 years (age less than 40 years: OR 0.21, 95% c.i. 0.07 to 0.61; age 40–49 years: OR 0.49, 95% c.i. 0.28 to 0.87) (see *Supplementary Table S2*). The use of ADM or mesh was independently associated with an increased risk of reoperation on (OR 2.77, 95% c.i. 1.15 to 6.68). In a sensitivity analysis excluding patients in which ADM was used, the effect of age on the risk of reoperation on was similar to the full analysis (age less than 40 years: OR 0.12, 95% c.i. 0.03 to 0.51; age 40–49 years: OR 0.52, 95% c.i. 0.29 to 0.94).

**Fig. 1:**
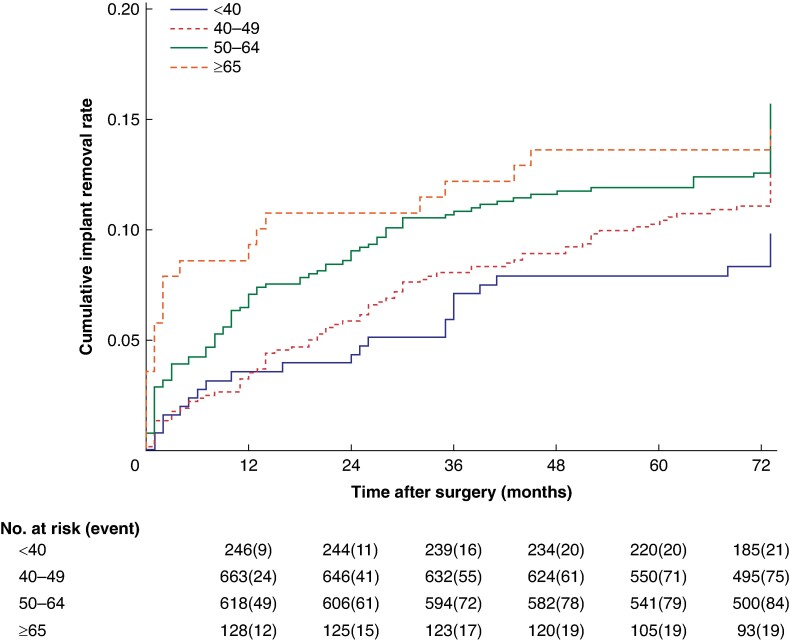
Cumulative implant removal rates for each age category Follow-up was truncated at 6 years for all patients.

By the end of follow-up, the implant or expander device had been removed in 266 patients. The 6-year probability of implant removal was 11.4% (*Fig. 1*). Of these 266 patients, 143 were converted to an autologous reconstruction either at the time of implant removal or later. The probability of implant removal at 6 years was not significantly different in the four age groups (less than 40 years: 8.7%, 40–49: 11.1%, 50–64: 13.5%, more than 65 years 14.3%, *P* = 0.217) even though a non-significant trend of increasing risk with higher age was observed. For women more than 65 years, implant removal occurred in 23 patients, 18 (78.3%) were due to a complication and the remainder due to patient preference. In five (21.7%) patients, a conversion to autologous reconstruction was undertaken either at the time of implant removal or in a later setting (3 due to patient preference and 2 due to complication). Implant removal within 30 days after surgery occurred in one patient less than 40 years (0.5%), four patients of 40–49 years (0.7%), eight of 50–64 years (1.6%) and four of more than 65 years (3.9%, *P* = 0.033). Older age was a risk factor for implant removal within 30 days on univariable regression analysis (OR 2.87, 95% c.i. 1.11 to 7.43) but not on multivariable analysis (OR 2.39, 95% c.i. 0.67 to 8.50).

## Discussion

The aim of the present study was to investigate 30-day postoperative complications and long-term surgical outcomes in breast cancer patients more than 65 years receiving implant-based IBR. While rates of postoperative infection did not differ across age groups, reoperation on within 30 days from IBR was most common in the oldest age group. Further, long-term implant removal rates did not differ across age groups. This study thus supports the notion that implant-based IBR is a safe surgical option in selected women more than 65 years. Future analyses should focus on the reasons for low IBR rates in this older age group with the aim to identify obstacles that can be addressed by improving patient selection strategies.

Patient selection for IBR is commonly based on a combined assessment of co-morbidities, disease characteristics and additional risk factors. While data on relevant co-morbidities were reliably captured, given that any surgery is preceded by an assessment by the anaesthetist documented in the medical chart, data on performance status were unfortunately not part of the data collection. When compared with previous publications such as those by Angarita *et al*. and Mays *et al.*, the population analysed in the present study had a significantly lower proportion of patients with antihypertensive medication (17.9% *versus* 64.1% and 58.7% respectively), which may suggest an even stricter selection^5,6^. Neither ischaemic heart disease (IHD) nor chronic obstructive pulmonary disease were captured in the present study, which may be a limitation since both conditions increase surgical risk.

Reported postoperative infection rates after implant-based IBR range from less than 1 to 43%, probably due to varying definitions of the term infection across different studies^22^. Francis *et al.* investigated rates and causes of postoperative infection after breast reconstruction using tissue expanders, using a similar definition of postoperative infection and reported rates comparable to the present results, namely 16.5%^23^.

In the present analysis, postoperative infection was not more common among older patients, which is in line with results from other reports^24–26^. On the other hand, a large cohort study by Angarita *et al.* focusing on 30-day postoperative complication rates in older women following implant-based and autologous IBR^27^ showed that women more than 70 years had a higher risk of postoperative infection than their younger counterparts (6% *versus* 4%). However, the definition of infection was not provided, which might explain contrary results^5^.

The present results suggest that women more than 65 years are not at a higher risk of reoperation on within 30 days following implant-based IBR. Instead, use of ADM or mesh was an independent risk factor for reoperation on within 30 days. This finding was not corroborated in a Swedish randomized clinical trial comparing implant-based IBR with and without the auxiliary use of ADM^28^. Even though rates of infections and reoperation on at 6 months and at 2 years were numerically higher in the ADM group, this finding was not statistically significant^28,29^. The mean age in that study was (mean(s.d.)) 51.8(9.5) years and 49.1(9.4) years in the respective groups, making it unlikely that a relevant number of women more than 65 years were included. The underrepresentation of older women is also evident in the randomized BRIOS^30^ trial that showed a higher risk of reoperation on in one-stage implant-based IBR using ADM compared with two-stage implant-based IBR without ADM^30^. The mean age was 43.5(11.7) years and 47.3(12.1) years respectively.

A major strength of the present study is the inclusion of all women with breast cancer undergoing implant-based IBR in Stockholm during an interval of 11 years, thus representing a population-based cohort. The long duration of the follow-up is important and was achieved by thorough individual review of medical records that resulted in high-quality data and completeness. On the other hand, any retrospective data collection is prone to selection bias, which may be especially relevant in the older age group: older women receiving IBR are most probably healthier than the average woman of the same age group and more often have low-risk disease, as mirrored by, for example the lower rates of adjuvant radiotherapy, which should be considered especially when comparing late complications such as implant removal. Furthermore, follow-up time for the oldest age group is shorter due to a shorter life expectancy, hence there is less time for a long-term complication such as implant removal to occur.

## Supplementary Material

zrae095_Supplementary_Data

## Data Availability

The authors are willing to make their data, analytic methods and study materials available to other researchers upon reasonable request, including relevant ethical and legal permissions to the corresponding author. The presented analysis was not preregistered.
