# Supplementary material for: Surgical complications after immediate implant-based breast reconstruction for breast cancer in women over 65 years
Source: BJS Open. 2024 Oct 9;8(5):zrae095. doi: 10.1093/bjsopen/zrae095 (PMC11462148; doi:10.1093/bjsopen/zrae095)
Supplement: zrae095_Supplementary_Data [file zrae095_supplementary_data.docx]

**Surgical complications after immediate implant-based breast reconstruction for breast cancer in women over 65**

Yihang Liu^1,2*^, Anna LV Johansson^3,4^, Ira Oikonomou^5^, Axel Frisell^6^, Hannah C Adam^7^, Dhirar Ansarei^8^, Martin Halle^1,9^, Helena Sackey^1,10^, Jana de Boniface^1,11^

^1^Department of Molecular Medicine and Surgery, Karolinska Institutet, Stockholm, Sweden

^2^Department of Acute and Trauma Surgery, Karolinska University Hospital, Stockholm, Sweden

^3^Department of Medical Epidemiology and Biostatistics, Karolinska Institutet, Stockholm, Sweden

^4^Cancer Registry of Norway, Oslo, Norway

^5^Department of Surgery, South General Hospital, Stockholm, Sweden

^6^Department of Dermatology and Venerology, Karolinska University Hospital, Stockholm, Sweden

^7^Department of Orthopaedic Surgery, Danderyd Hospital, Stockholm, Sweden

^8^Department of Surgery, Västmanlands sjukhus Västerås, Västerås, Stockholm, Sweden

^9^Department of Reconstructive Plastic Surgery, Karolinska University Hospital, Stockholm, Sweden

^10^Department of Breast Cancer, Endocrine Tumours and Sarcoma, Karolinska University Hospital, Stockholm, Sweden

^11^Department of Surgery, Capio St Göran’s Hospital, Stockholm, Sweden

*Correspondence to: Yihang Liu, Department of Acute and Trauma Surgery, Karolinska University Hospital Huddinge, Hälsovägen 13, SE-14157 Stockholm, Sweden

**Supplementary Materials - Index**

|  |  |
| --- | --- |
| **Supplementary Figures and Tables** |  |
| Supplementary table 1. Factors associated with postoperative infection within 30 days; uni- and multivariable logistic regression analysis | *page 3-4* |
| Supplementary table 2. Factors associated with reoperation within 30 days; uni- and multivariable logistic regression analysis. | *page 5-7* |
|  |  |

Supplementary table 1. Factors associated with postoperative infection within 30 days; uni- and multivariable logistic regression analysis.

|  |  | **Univariable** | | **Multivariable^1^** | |
| --- | --- | --- | --- | --- | --- |
|  | **N (events)** | **OR (95% CI)** | **P-value** | **OR (95% CI)** | **P-value** |
| **Age groups (years)** |  |  |  |  |  |
| Under 40 | 218 (37) | 0.74 (0.49-1.11) | 0.149 | 0.85 (0.54-1.34) | 0.495 |
| 40-49 | 562 (97) | 0.75 (0.55-1.02) | 0.069 | 0.83 (0.60-1.15) | 0.265 |
| 50-64 | 493 (107) | 1.00 (ref) |  | 1.00 (ref) |  |
| ≥65 | 102 (15) | 0.62 (0.34-1.12) | 0.114 | 0.63 (0.34-1.14) | 0.127 |
| **Calendar interval for IBR** |  |  |  |  |  |
| 2005-2007 | 197 (35) | 1.00 (ref) |  | 1.00 (ref) |  |
| 2008-2010 | 380 (69) | 1.03 (0.66-1.61) | 0.908 | 1.00 (0.62-1.59) | 0.990 |
| 2011-2013 | 451 (90) | 1.15 (0.75-1.78) | 0.516 | 1.20 (0.77-1.93) | 0.397 |
| 2014-2015 | 347 (62) | 1.00 (0.64-1.59) | 0.976 | 1.02 (0.60-1.71) | 0.947 |
| **Previous radiotherapy** |  |  |  |  |  |
| No previous RT | 1274 (233) | 1.00 (ref) |  | 1.00 (ref) |  |
| Previous RT | 101 (23) | 1.32 (0.81-2.14) | 0.266 | 1.23 (0.70-2.18) | 0.475 |
| **Axillary surgery** |  |  |  |  |  |
| No axillary surgery | 86 (18) | 1.00 (ref) |  | 1.00 (ref) |  |
| Sentinel node biopsy only | 757 (145) | 0.89 (0.52-1.55) | 0.693 | 0.96 (0.51-1.80) | 0.891 |
| Axillary lymph node dissection | 532 (93) | 0.80 (0.45-1.41) | 0.440 | 0.86 (0.44-1.67) | 0.655 |
| **Body Mass Index (kg/m^2^)** |  |  |  |  |  |
| Underweight (<18.5) | 28 (2) | 0.37 (0.09-1.58) | 0.180 | 0.40 (0.09-1.74) | 0.222 |
| Normal weight (18.5-24.9) | 874 (150) | 1.00 (ref) |  | 1.00 (ref) |  |
| Pre-obesity (25.0-29.9) | 391 (81) | 1.26 (0.93-1.70) | 0.131 | 1.04 (0.74-1.45) | 0.835 |
| Obesity (≥30.0) | 82 (23) | 1.88 (1.13 -3.14) | 0.016 | 1.37 (0.77-2.43) | 0.281 |
| **Nipple-sparing mastectomy** |  |  |  |  |  |
| No | 1187 (213) | 1.00 (ref) |  | 1.00 (ref) |  |
| Yes | 188 (43) | 1.36 (0.94-1.97) | 0.108 | 1.52 (1.00-2.32) | 0.050 |
| **Mastectomy specimen weight categories (g)** |  |  |  |  |  |
| < 300 | 551 (78) | 1.00 (ref) |  | 1.00 (ref) |  |
| 300-500 | 495 (99) | 1.52 (1.09 -2.10) | 0.012 | 1.86 (1.27-2.72) | 0.001 |
| > 500 | 329 (79) | 1.92 (1.35-2.71) | <0.001 | 2.37 (1.50-3.72) | <0.001 |
| **Matrix use** |  |  |  |  |  |
| No | 1312 (234) | 1.00 (ref) |  | 1.00 (ref) |  |
| Yes (ADM or synthetic mesh) | 63 (22) | 2.47 (1.44-4.23) | <0.001 | 2.04 (1.10-3.76) | 0.023 |
| **Smoking status** |  |  |  |  |  |
| Non-smoker | 1101 (186) | 1.00 (ref) |  | 1.00 (ref) |  |
| Active smoker | 126 (39) | 2.20 (1.46-3.32) | <0.001 | 2.25 (1.47-3.46) | <0.001 |
| Previous smoker | 148 (31) | 1.30 (0.85-2.00) | 0.223 | 1.23 (0.79-1.91) | 0.362 |
| **Antihypertensive medication** |  |  |  |  |  |
| No | 1287 (235) | 1.00 (ref) |  | 1.00 (ref) |  |
| Yes | 88 (21) | 1.40 (0.84-2.34) | 0.193 | 1.23 (0.71-2.13) | 0.460 |
| **Type of breast implant or expander device** |  |  |  |  |  |
| Permanent implant | 322 (81) | 1.38 (0.98-1.93) | 0.062 | 1.74 (1.16-2.61) | 0.007 |
| Permanent expander | 642 (106) | 1.00 (ref) |  | 1.00 (ref) |  |
| Temporary expander | 411 (69) | 1.24 (0.90-1.71) | 0.186 | 1.18 (0.84-1.65) | 0.348 |
| **Primary treatment** |  |  |  |  |  |
| Primary surgery | 1185 (224) | 1.00 (ref) |  | 1.00 (ref) |  |
| Neoadjuvant chemotherapy | 190 (32) | 0.87 (0.58-1.30) | 0.498 | 0.97 (0.60-1.58) | 0.915 |

^1^ Model adjusted for all variables in the table.

IBR; immediate breast reconstruction. RT; radiotherapy. ADM (Acellular dermal matrix)

Supplementary table 2. Risk factors associated with reoperation within 30 days; uni- and multivariable logistic regression analysis.

|  |  | **Univariable** | | **Multivariable^1^** | |
| --- | --- | --- | --- | --- | --- |
|  | **N (events)** | **OR (95% CI)** | **P-value** | **OR (95% CI)** | **P-value** |
| **Age (years)** |  |  |  |  |  |
| <40 | 218 (4) | 0.24 (0.08-0.67) | 0.007 | 0.21 (0.07-0.61) | 0.004 |
| 40-49 | 561 (22) | 0.52 (0.30-0.89) | 0.018 | 0.49 (0.28-0.87) | 0.015 |
| 50-64 | 492 (36) | 1.00 (ref) |  | 1.00 (ref) |  |
| ≥65 | 102 (8) | 1.08 (0.49-2.39) | 0.854 | 1.00 (0.44-2.28) | 0.993 |
| **Calendar interval for IBR** |  |  |  |  |  |
| 2005-2007 | 197 (5) | 1.00 (ref) |  | 1.00 (ref) |  |
| 2008-2010 | 380 (19) | 2.02 (0.74-5.50) | 0.168 | 2.00 (0.72-5.57) | 0.182 |
| 2011-2013 | 450 (21) | 1.88 (0.70-5.06) | 0.212 | 1.84 (0.67-5.10) | 0.239 |
| 2014-2015 | 346 (25) | 2.99 (1.13-7.42) | 0.028 | 2.38 (0.83-6.85) | 0.108 |
| **Previous radiotherapy** |  |  |  |  |  |
| No previous RT | 1273 (66) | 1.00 (ref) |  | 1.00 (ref) |  |
| Previous RT | 100 (4) | 0.76 (0.27-2.13) | 0.605 | 0.45 (0.14-1.44) | 0.178 |
| **Axillary surgery** |  |  |  |  |  |
| No axillary surgery | 86 (5) | 1.00 (ref) |  | 1.00 (ref) |  |
| Sentinel node biopsy only | 755 (42) | 0.95 (0.37-2.48) | 0.924 | 0.73 (0.25-2.13) | 0.570 |
| Axillary lymph node dissection | 532 (23) | 0.73 (0.27-1.98) | 0.539 | 0.64 (0.20-2.02) | 0.443 |
| **Body Mass Index (kg/m^2^)** |  |  |  |  |  |
| Underweight (<18.5) | 28 (2) | 1.64 (0.38-7.18) | 0.508 | 1.90 (0.41-8.77) | 0.412 |
| Normal weight (18.5-24.9) | 873 (39) | 1.00 (ref) |  | 1.00 (ref) |  |
| Pre-obesity (25.0-29.9) | 390 (23) | 1.34 (0.79-2.28) | 0.279 | 1.22 (0.67-2.21) | 0.519 |
| Obesity (≥30.0) | 82 (6) | 1.69 (0.69 -4.11) | 0.249 | 1.53 (0.56-4.15) | 0.407 |
| **Nipple-sparing mastectomy** |  |  |  |  |  |
| Yes | 188 (59) | 1.19 (0.61-2.30) | 0.614 | 1.17 (0.56-2.46) | 0.675 |
| No | 1185 (11) | 1.00 (ref) |  | 1.00 (ref) |  |
| **Mastectomy specimen weight categories (g)** |  |  |  |  |  |
| < 300 | 551 (24) | 1.00 (ref) |  | 1.00 (ref) |  |
| 300-500 | 495 (24) | 1.20 (0.63 -2.00) | 0.704 | 1.34 (0.68-2.63) | 0.394 |
| > 500 | 327 (22) | 1.58 (0.87-2.87) | 0.130 | 1.74 (0.79-3.83) | 0.167 |
| **Matrix use** |  |  |  |  |  |
| No | 1310 (61) | 1.00 (ref) |  | 1.00 (ref) |  |
| Yes (ADM or synthetic mesh) | 63 (9) | 3.41 (1.61-7.23) | 0.001 | 2.77 (1.15-6.68) | 0.023 |
| **Smoking status** |  |  |  |  |  |
| Non-smoker | 1099 (56) | 1.00 (ref) |  | 1.00 (ref) |  |
| Active smoker | 126 (6) | 0.93 (0.39-2.21) | 0.871 | 0.85 (0.35-1.92) | 0.731 |
| Previous smoker | 148 (8) | 1.06 (0.50-2.28) | 0.873 | 0.89 (0.41-1.95) | 0.775 |
| **Antihypertensive medication** |  |  |  |  |  |
| Yes | 88 (5) | 1.31 (0.44-2.88) | 0.797 | 0.69 (0.26-1.84) | 0.456 |
| No | 1285 (65) | 1.00 (ref) |  | 1.00 (ref) |  |
| **Type of breast implant or expander device** |  |  |  |  |  |
| Permanent implant | 322 (20) | 1.08 (0.62-1.89) | 0.790 | 1.18 (0.60-2.31) | 0.625 |
| Permanent expander | 640 (37) | 1.00 (ref) |  | 1.00 (ref) |  |
| Temporary expander | 411 (13) | 0.53 (0.28-1.01) | 0.055 | 0.61 (0.31-1.18) | 0.144 |
| **Primary treatment** |  |  |  |  |  |
| Primary surgery | 1183 (62) | 1.00 (ref) |  | 1.00 (ref) |  |
| Neoadjuvant chemotherapy | 190 (8) | 0.79 (0.37-1.69) | 0.550 | 1.19 (0.49-2.91) | 0.705 |

^1^ Model adjusted for all variables in the table.

IBR; immediate breast reconstruction. RT; radiotherapy. ADM (Acellular dermal matrix)
